# Molecular and Supramolecular Changes in Polybutylene Succinate (PBS) and Polybutylene Succinate Adipate (PBSA) Copolymer during Degradation in Various Environmental Conditions

**DOI:** 10.3390/polym10030251

**Published:** 2018-03-01

**Authors:** Michał Puchalski, Grzegorz Szparaga, Tadeusz Biela, Agnieszka Gutowska, Sławomir Sztajnowski, Izabella Krucińska

**Affiliations:** 1 Faculty of Material Technologies and Textile Design, Department of Material and Commodity Sciences and Textile Metrology, Centre of Advanced Technologies of Human-Friendly Textiles “Pro Humano Tex”, Lodz University of Technology, Zeromskiego 116, 90-924 Lodz, Poland; grzegorz.szparaga@p.lodz.pl (G.S.); slawomir.sztajnowski@p.lodz.pl (S.S.); izabella.krucinska@p.lodz.pl (I.K.); 2 Centre of Molecular and Macromolecular Studies, Polish Academy of Sciences, Sienkiewicza 112, 90-301 Lodz, Poland; tadek@cbmm.lodz.pl; 3 Institute of Security Technologies “MORATEX”, Sklodowskiej-Curie 3, 90-505 Lodz, Poland; agutowska@moratex.eu; 4 Department of Synthetic Fibers, Institute of Biopolymers and Chemical Fibres, Skłodowskiej-Curie 19/27, 90-570 Lodz, Poland

**Keywords:** PBS, PBSA, WAXD, SEC-MALLS, degradation, composting, artificial weathering

## Abstract

In this paper, the influence of the various degradation conditions, on the molecular and supramolecular structure of polybutylene succinate (PBS) and polybutylene succinate adipate (PBSA) copolymer during degradation is described. The experiment was carried out by the use of injection molded samples and normalized conditions of biodegradation in soil, composting and artificial weathering. Materials were studied by size-exclusion chromatography (SEC) coupled with multiangle laser light scattering (MALLS) detection and wide-angle X-ray diffraction (WAXD). Additionally, the physical and mechanical properties of the samples were determined. The performed experiments clearly show difference impacts of the selected degradation conditions on the macroscopic, supramolecular and molecular parameters of the studied aliphatic polyesters. The structural changes in PBS and PBSA explain the observed changes in the physical and mechanical properties of the obtained injection molded samples.

## 1. Introduction

In recent years, pro-ecological trends in waste reduction have led to the substitution of durable polymers with biodegradable polymers. For this, thermoplastic aliphatic polyesters are promising alternatives [1,2]. In this group of polymers, polybutylene succinate (PBS) is an interesting material from an application standpoint, as its mechanical properties are similar to those of popular polymers, such as polypropylene (PP) [3].

Polybutylene succinate (PBS) is a biodegradable aliphatic polyester produced by the polycondensation of succinic acid (SA) and 1,4-butanediol (BD) [4]. The synthesis and characterization of PBS and polybutylene adipate (PBA), which is synthesized from 1,4-butanediol (BD) and adipate acid (AA), are extensively described in the literature using mathematical models [5,6]. Due to the high crystallinity and good thermal properties of the homopolymers, a copolymer of PBS and PBA has been used in selected applications, such as packaging [7]. The physical properties and crystalline structure of the polybutylene succinate adipate (PBSA) copolymer strongly depend on the composition of the polymer compounds. Moreover, the PBSA copolymer is characterized by a higher degradability by enzymatic processes compared with other materials due to its lower crystallinity [8].

For many years, PBS and PBSA have been produced from petrochemical sources by Showa Highpolymer (Shanghai, China). The polymer, which has the trade name Bionolle, is characterized by its similar processability to that of conventional resins, such as polyethylene. Bionolle is one of the most suitable materials for processing into films, which can then be utilized for agricultural purposes, shopping bags, compost bags, and so on. Showa Highpolymer has succeeded in producing a compound of Bionolle and starch that not only has similar properties to homogeneous Bionolle but is also environmentally friendly [9,10,11].

The lack of renewability and the rising price of fossil resources have limited the use of petrochemical succinic acid for a wide range of applications. Consequently, the natural next step was to develop a synthetic method for bio-succinic acid from a renewable source, such as biomass. The yeast- or bacteria-based production of succinic acid and the possibility of sourcing 1,4-butanediol from ecological methods make PBS and its copolymers attractive biodegradable polymers that are completely produced from renewable resources [12,13,14].

Currently, research on this topic is related to the search for new applications of PBS, for example, the development of novel materials for ecological agriculture purposes. Developed materials, such as mulching nonwovens and pots produced from nonwovens, are interesting alternatives to polypropylene products [15,16]. Another area of work is the use of polybutylene succinate as an additive to plasticize other biodegradable polymers, such as polylactide (PLA) [17,18,19]. Research is therefore concerned with the search for new applications for this aliphatic polyester [20,21,22].

On the other hand, research has focused on the analysis of the degradation of PBS, PBA and their copolymers by the examination of new degradation conditions, enzymes and microorganisms [8,23,24,25]. This topic is still developing and continues to expand the understanding of this polymer and other aliphatic polyesters. The impacts of the degradation process on weight loss, the decrease in the molar mass and changes in mechanical properties have been evaluated [26,27]. The kinetics of the degradation process have also been analyzed [28]. However, there are few results for the assessment of changes in the supramolecular structure during the PBS degradation process [29]. For other aliphatic polyesters (e.g., PLA), such changes have been observed and were responsible, inter alia, for a decrease in the mechanical properties [30]. The influences of properties and structures of aliphatic polyesters and their copolymers, such as molar mass, thermal properties, degree of crystallinity or the ratio between ester bonds on their ability to degrade, were also analyzed [31,32,33,34].

In this study, the degradation of a commercially available PBS and PBSA copolymer in three various regimes—biodegradation in compost, biodegradation in soil and artificial weathering—was performed. The experiment was carried out under laboratory conditions according to the obligatory standard. For complete analysis, the change in the studied samples during degradation was analyzed on the macroscopic, supramolecular and molecular scales. The estimation of mass loss and change in the mechanical properties was carried out according to the obligatory standards, while supramolecular and molecular parameters were investigated by using wide-angle X-ray diffraction (WAXD) and size-exclusion chromatography (SEC) coupled with multiangle laser light scattering (MALLS) detection.

## 2. Materials and Methods

### 2.1. Materials

In the experiments, the commercially available polybutylene succinate (PBS) Bionolle 1020 MD and the polybutylene succinate adipate (PBSA) copolymer Bionolle 3020 MD were purchased from Showa Denko K.K. (Tokyo, Japan). For the tensile test, the samples were formed into dog-bone shaped specimens by the use of an injection molding machine (Allrounder 420C, Arburg, Loßburg, Germany) according to the ISO-527-2-1A standard. The polymer materials were formed at 180 °C (PBS, 1020 MD) and 170 °C (PBSA, 3020 MD).

### 2.2. Degradation Environment

#### 2.2.1. Laboratory Biodegradation

The laboratory biodegradation of the PBS and PBSA samples was conducted under controlled conditions in accordance with the International Standard, PN-EN ISO 20200:2016, and European Standards, PN-EN 14806:2010 and PN-EN 14045:2005. The experiment was carried out using common commercially available garden soil with a pH of 6.0–6.5 and 1.8 × 10^7^ cfu/g microorganisms and compost from an industrial compost prism (Municipal Services Company of the city of Łódź, Łódź, Poland) with a pH of 7 and 3.2 × 10^7^ cfu/g microorganisms [15,35]. The studied samples were biodegraded in the garden soil at a temperature of 30 ± 2 °C, and the medium had a moisture content of 55.6%, while the samples were biodegraded in compost at a temperature of 58 ± 2 °C, and the medium had a moisture content of 53.1%, which was in line with the adopted standards. The biodegradation processes were controlled to have defined time intervals (1, 4, 8, 12, 16, 20 and 24 weeks). During the experiment, moisture was replenished to the initial value with water. After a defined incubation time, the samples were dried to a constant weight, and the mass loss was estimated. Each sample was tested 3 times. The resulting variation in the results of the biodegradation tests was below 10%.

#### 2.2.2. Laboratory Artificial Weathering

The laboratory artificial weathering of the studied PBS and PBSA samples was carried out in a QUV chamber, in accordance with the standard, PN-EN ISO 4892-3, based on the Technical Report TR 010 ed. May 2004 “Exposure procedure for artificial weathering”. The weathering process was performed in cycles, irradiating the samples with a light intensity of 0.76 W/m^2^ by UVA-340 nm radiation under the following conditions:

- 8 h of exposure—chamber temperature (ChT) 50 °C, relative humidity (RH) 40%;
- 4 h of artificial rainfall with demineralized water and no exposure—ChT = 20 °C.

The samples were weathered for defined time intervals—45, 90, 180, 360, 540, 720, 900, 1080, 1260, 1440 and 1800 h—where the 720 h interval corresponded to the natural exposure of a sample over 1 year in a moderate climate (e.g., in Poland).

### 2.3. Mechanical Properties

The mechanical parameters of the PBS and PBSA samples that did not fall apart during degradation were measured using an Instron 5511 mechanical testing machine (Instron, Canton, MA, USA). The tests were carried out according to the PN-EN ISO 527-2:2012 standard.

### 2.4. SEC-MALLS Method

The molar mass (*M*_n_) and dispersity (*Đ*) of the studied PBS and PBSA materials were analyzed by size-exclusion chromatography (SEC) coupled with multiangle laser light scattering (MALLS) detection. The SEC−MALLS instrument was composed of an Agilent 1100 isocratic pump, an auto-sampler, a degasser, a thermostatic box for columns, a MALLS DAWN HELEOS-II photometer (Wyatt Technology Corporation, Santa Barbara, CA, USA) and an Optilab T-rEX differential refractometer. The ASTRA 4.90.07 software package (Wyatt Technology Corporation) was used for data collection and processing. Two PLGel 5 μm MIXD-C columns were used for separation. The samples were injected as a methylene chloride solution. The volume of the injection loop was 100 μm^3^. Methylene chloride was used as the mobile phase at a flow rate of 0.8 cm^3^·min^−1^. The calibration of the DAWN HELEOS-II photometer was performed using p.a. grade toluene, and normalization was performed using a polystyrene standard (*M*_n_ = 30,000 g/mol). The measurements were conducted at room temperature.

### 2.5. WAXS Method

The changes in the supramolecular structures of the PBS and PBSA samples during degradation were investigated by wide-angle X-ray diffraction scattering (WAXS) using an X’Pert PRO diffractometer (CuKα source, λ = 0.154 nm) from PANalytical (Almelo, The Netherlands). The X-ray diffractograms were recorded in the 2θ range of 5° to 60° with a step of 0.05°. The obtained WAXS data were analyzed numerically using the WAXSFIT software (2.0, University of Bielsko Biała, Bielsko-Biała, Poland) [36].

## 3. Results and Discussion

### 3.1. Analysis of the Physical Properties of the Studied Materials after Degradation

**Figure 1:**
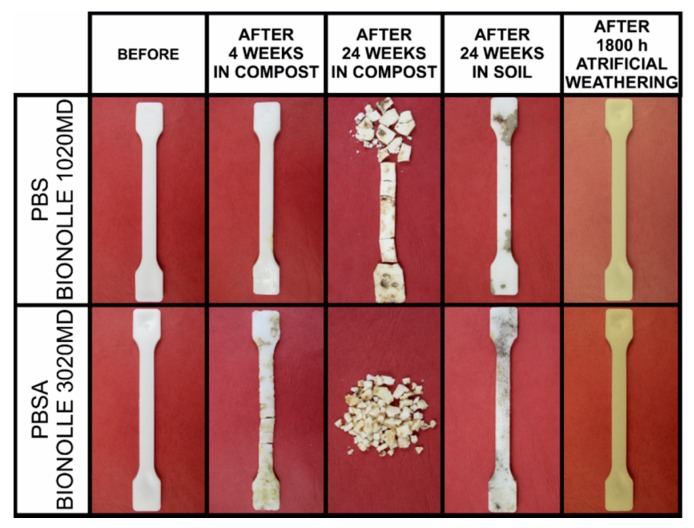
Photographs of the investigated samples before and after degradation.

The physical changes in the structures of the studied materials were directly observed and recorded photographically. In Figure 1, a collection of selected photographs of the degradation processes occurring in various conditions is presented. According to these photographs, the most degrading medium was compost. During PBSA biodegradation in compost, the tested sample began to fragment after 4 weeks, and PBS began to fragment after 6 weeks. The other degradation conditions applied in the investigation did not result in fragmentation of the studied materials. Matting of the samples’ surfaces and additional color changes are the only visible changes resulting from artificial weathering.

**Figure 2:**
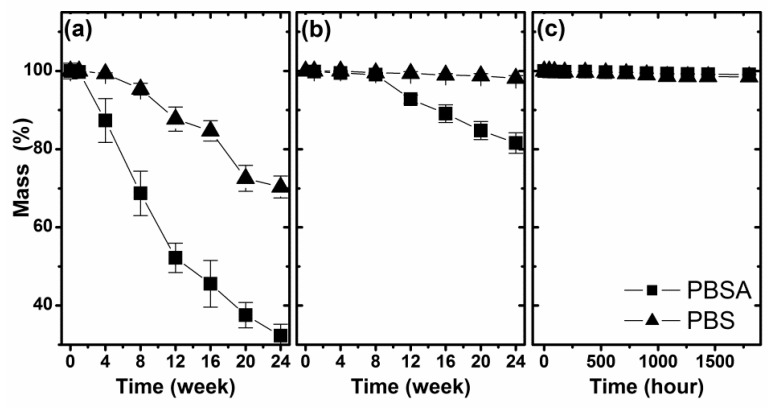
The mass changes of the studied samples during degradation in selected environments: (**a**) biodegradation in compost, (**b**) biodegradation in soil and (**c**) artificial weathering.

The most often analyzed macroscopic factor in material degradation assessments is mass loss. In Figure 2, the mass changes of the studied PBS and PBSA samples as a function of degradation time are shown. The most visible mass loss was measured for the samples biodegraded in compost. (Figure 2a), while the weakest initiator of mass loss was artificial weathering, where the maximum mass loss was only around 1% for both studied materials (Figure 2c). The insignificant changes in the sample mass during aging under simulated atmospheric conditions seems to be a result of the absence of its direct physical contact with the degrading medium. In the soil or in compost, the degraded products probably diffuse from the surface layer into the environment, which is observed as a decrease in mass. Furthermore, in compost, a visible strong mass loss also results from the reaction with enzymes that support the erosion of the surface of the studied material.

Analysis of the difference between both studied Bionolle polymers clearly shows a higher mass loss from the PBSA material than the PBS material over the same time period. It is worth noting that the PBS polymer lost a significant amount of mass only during biodegradation in compost, and other initiators did not cause a decrease in its mass.

**Figure 3:**
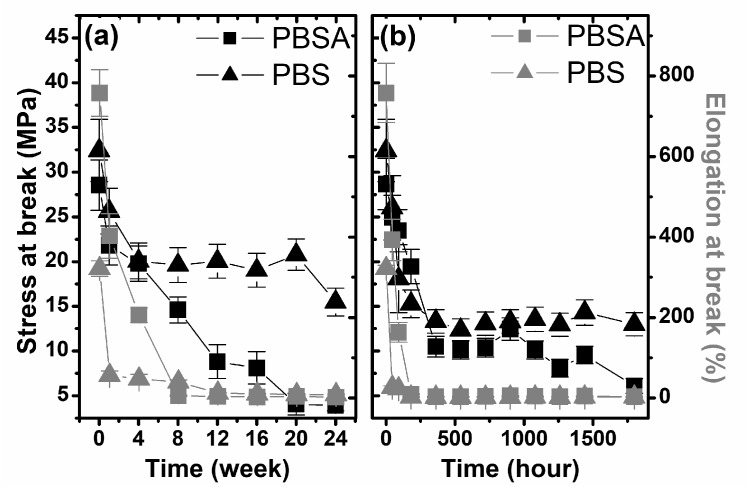
The changes in the mechanical properties of the studied samples during degradation in selected environments: (**a**) biodegradation in soil and (**b**) artificial weathering.

The next step in the macroscopic analysis of physical changes of the studied samples was the investigation of their mechanical properties. In Figure 3, the stress and elongation at break-point as a function of degradation time is shown. As it is seen, there is a lack of information about changes in the mechanical properties of the studied samples during biodegradation in compost. Samples that were composted for more than 1 or 4 weeks were fragmented into smaller pieces, which prevented mechanical testing (Figure 1). The stress and elongation at break-point of PBS after 4 weeks (maximum time after which the sample is not fragmented) decreased from 34.8 MPa to 12.7 MPa and 321.8% to 1.7% respectively, while for PBSA, after 1 week, these values decreased from 28.6 MPa to 21.8 MPa and from 757.9% to 196.1%. These results indicate the strong degradation of both polymers in compost, which is probably an effect of its composition being more complex than soil from a microbiological (enzymatic) point of view. The changes in the mechanical properties of the studied polymers as a function of degradation time clearly show a large decrease in the elasticity of the materials during the degradation process, both in the biodegradation process in soil and in the irradiation process of artificial weathering, as presented in Figure 3. Furthermore, as a result of the artificial weathering process, the strength of both studied polymers was significantly reduced (Figure 3b). Considering the lack of mass loss and matting of the sample surface, it can be concluded that the structure of the polymers changed on a molecular and a supramolecular level, as discussed in the next part of the paper.

Slightly different changes in the stress of the polymers were observed in the biodegradation process in soil (Figure 3a). For both PBS and PBSA, the rates of change were lower than during degradation under artificial weathering conditions. However, in the case of a polymer containing an adipate functional group, the strength of the tested samples decreased considerably with the time of degradation and after 24 weeks, was at a level of 4 MPa. Based on the analysis of the estimated macroscopic factors, it can be concluded that the most degrading environment is compost. This could be expected, because in the compost degradation process, carried out in accordance with the ISO standard, the degradation of polymers occurs at least for two processes: hydrolysis, which is supported by moisture in the medium [29], and biodegradation, which is realized by natural enzymes and microorganisms [37,38]. Less intense effects of polymer degradation were obtained using soil, which is the result of the lower content of microorganisms and enzymes as well as the realizing of the process at lower temperatures, in accordance with the ISO standard used, compared to the case of degradation in compost. Notably, both of applied degradation conditions showed greater susceptibility to degradation of the polymer containing the adipate functional group, which is consistent with the current state of knowledge [34].

### 3.2. Changes in the Molar Mass and Dispersity of the Studied Materials during Degradation

**Figure 4:**
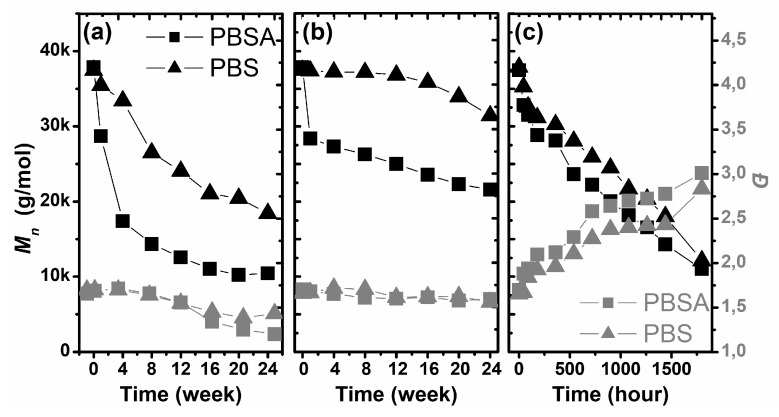
Changes in molar mass (*M*_n_) (black symbols) and dispersity (gray symbols) of the studied samples during degradation in selected environments: (**a**) biodegradation in compost, (**b**) biodegradation in soil and (**c**) artificial weathering.

The molecular parameters (*M*_n_ and *M*_w_/*M*_n_ = *Đ*) of the studied polymers were analyzed using the SEC-MALLS method. In Figure 4, the number average molar mass (*M*_n_) and dispersity (*Ð*) [39] of the studied samples as a function of degradation time under the selected conditions are presented. The most visible change in the molecular parameters of the studied polymers was observed for the samples degraded by the artificial weathering process. Exposure of the sample to ultra violet (UV) radiation, high relative humidity and heat resulted in a decrease in the number average molar weight and an increase in the dispersity of both studied polymers (Figure 4c). During this process, the value of *M*_n_ decreased from 37.4 kg/mol and 37.8 kg/mol, to 12.1 kg/mol and 11.0 kg/mol, for PBS and PBSA, respectively. However, the dispersity increased from 1.65–1.69 to 2.8–3.0. The obtained results suggest the strong influence of artificial weathering on the molecular parameters of both polymers.

The number—average molar mass dependence on the degradation time during artificial weathering was almost linear. A similar linear tendency was also observed for the dispersity, but this molecular parameter increased with degradation time. Artificial weathering resulted in a random process for the decomposition of PBS and PBSA macromolecules. Furthermore, the obtained results corresponded to the estimated macroscopic factors of degradation, and suggested a lack of significant influence of addition of the adipate functional group to PBS on the degradation of PBS during artificial weathering.

Obviously, different results were obtained for the biodegradation of both polymers in compost: the polymer molecular weight decreased, accompanied by a reduction in the dispersity. The loss of Mn as a result of degradation is a typical phenomenon in this process, but the decreased dispersity suggests another degradation mechanism than that observed for artificial weathering. For up to 12 weeks, the dispersity was approximately 1.60 for both studied polymers; however, during the next interval of biodegradation, an insignificant decrease was observed, and finally, after 24 weeks, the dispersities of PBS and PBSA were 1.43 and 1.20, respectively. This confirms at least two mechanisms to be operating during the biodegradation in compost: the relatively slow hydrolysis of higher molar mass polymers and the faster enzymatic etching of oligomers. It is important to emphasize that enzymes are capable of etching only polymers with a sufficiently low molar mass. Therefore, this process is not random, as is the degradation during artificial weathering. Hydrolysis leads to a decrease in the overall molar mass, but enzymatic etching eliminates the oligomeric fraction from the samples relatively quickly. As a consequence, both the molar mass and dispersity are reduced. Additionally, the decrease in *M*_n_ as a function of degradation time was non-linear and showed an almost exponential decrease. Moreover, the obtained results showed that the PBSA copolymer was more resistant to degradation in compost than the PBS polymer.

The degradation process was also analyzed in soil (Figure 4b). Biodegradation of the studied polymers in soil gave similar results to the biodegradation in compost, but the changes in the molecular parameters were less intense, which is an effect of the lower content of microorganisms and lower temperature of the process. The *M*_n_ decreased, but without significant changes in dispersity, which indicates the random degradation of polymer chains, with uniform molecular weight losses in all polymer fractions. At the end of the experiment, the *M*_n_ values of PBS and PBSA were 31 and 23 kg/mol, respectively. As is clearly shown, PBSA was more resistant to degradation in soil, and a visible decrease in the molar mass was observed only after 16 weeks. The profile of the molar mass loss of PBS as a function of degradation time was similar to that for biodegradation in compost. The main visible change in *M*_n_ was observed only during the initial period of biodegradation, up to 4 weeks.

### 3.3. Analysis of the Supramolecular Structure of the Studied Materials after Degradation

**Figure 5:**
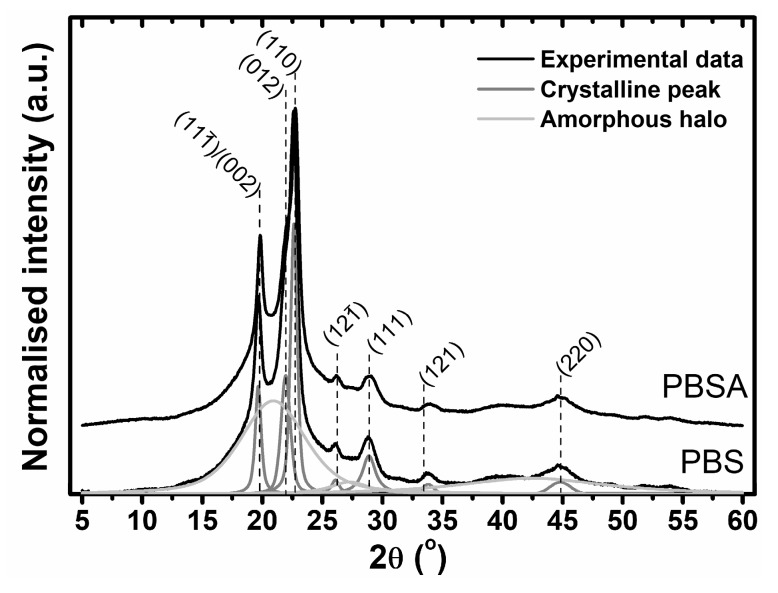
X-ray diffraction profiles of PBS and PBSA samples with deconvolution to the amorphous and crystalline compounds.

The presented SEC-MALLS results showed the influence of various conditions on the mechanism of polymer degradation and on the changes in the molecular structures of the studied polymers. To estimate the changes in the supramolecular structures of the materials during degradation, wide-angle X-ray diffraction was employed. In Figure 5, examples of the X-ray diffraction profiles of both studied polymers are shown. The X-ray diffraction peaks at 2θ = 19.7°, 22.1°, 22.8°, 26.2° and 29.1°, corresponding to the (111̅)/(002), (012), (110), (121̅) and (111) planes of the poly(butylene) succinate monoclinic crystal lattice, were clearly visible. The diffraction patterns do not give much information about the poly(butylene) adipate crystalline structure. The strongest diffraction peaks at 2θ = 17.6° and 21.7°, corresponding to the (002) and (110) planes of PBA, are not detectable. This result indicates that a low content (<25 wt %) of poly(butylene) adipate is present in the studied copolymer [22].

Due to slight changes in the X-ray profiles of the polymers as a result of the degradation process, numerical analysis of the profiles was performed to estimate the quantitative influence of degradation on the supramolecular structures of the studied materials. For this analysis, deconvolution of the experimental data to the amorphous halo and crystalline peaks was performed in accordance with Hindeleh and Johnson’s method (Figure 5). The X-ray diffraction patterns were directly analyzed to obtain the crystalline phase content, using Equation (1):

(1) $$\chi_{C}=\frac{A_{C}}{A_{C}+A_{A}}$$

where *A*_A_ and *A*_C_ are the integrated areas calculated under the amorphous and crystalline curves, respectively.

**Figure 6:**
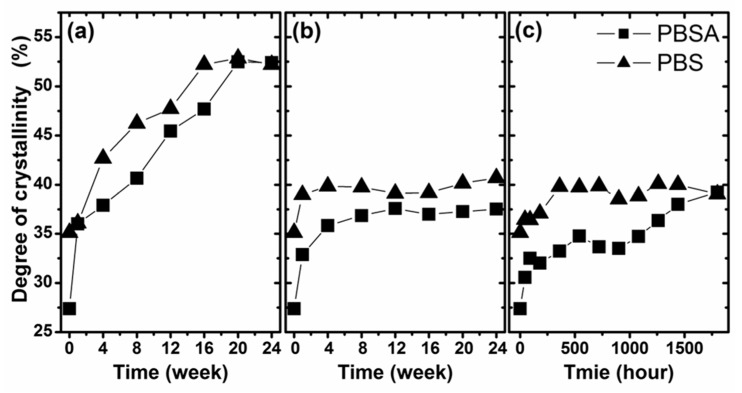
Changes in the crystallinity of the studied samples during degradation in selected environments: (**a**) biodegradation in compost, (**b**) biodegradation in soil and (**c**) artificial weathering.

In Figure 6, the degree of crystallinity of the studied polymers as a function of degradation time under various conditions is presented. As is clearly shown, the largest change in the crystallinity was observed after biodegradation in compost. The degree of crystallinity (35% for PBS and 27% for PBSA) increased to 52% for both materials. Notably, during biodegradation in compost, the polymer crystallized almost linearly. This phenomenon was not observed in the other tested degradation conditions, where the degree of crystallinity increased significantly in the initial period and then did not change significantly. The maximum degree of crystallinity for PBS was approximately 40% in both conditions, while for PBSA, this value was approximately 37% for biodegradation in soil and approximately 40% for artificial weathering. The presented results of the change in crystallinity correlate with the results presented above, in regard to which compost is the most degrading environment for the studied polymers. The increase of crystallinity is a result of the hydrolysis and enzymatic degradation where amorphous parts of polymers are degraded first, followed by crystalline. Furthermore, the differences between the studied polymers can be attributed to the different degree of crystallinity of the studied materials as well as due to the different crystalline properties [40]. The increase in the degree of crystallinity during degradation also affords an understanding of the observed macroscopic changes in the studied samples. The high crystallinity of the samples after biodegradation in compost explains their ability to fragment. Mechanical tests could be carried out for materials with lower crystallinity, i.e., biodegraded in soil and aged by artificial weathering.

**Figure 7:**
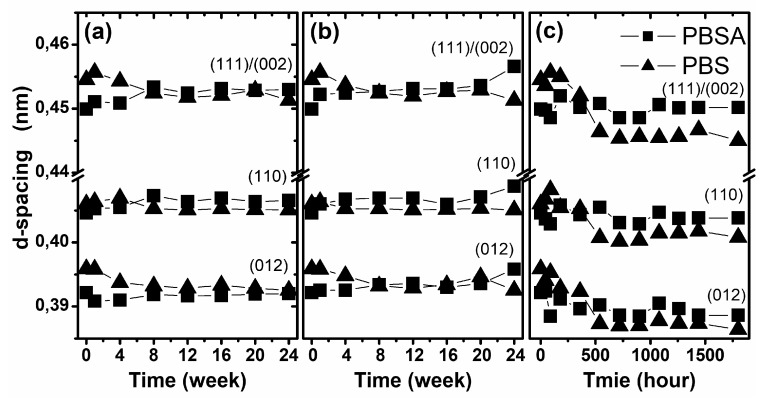
Changes in the d-spacing of the studied samples during degradation in selected environments: (**a**) biodegradation in compost, (**b**) biodegradation in soil and (**c**) artificial weathering.

Another characteristic feature of the structure of the studied polymers degraded under different conditions is the lattice length (d-spacing), which can be calculated using Braggs Equation (2):

(2) $$d=\frac{\lambda }{2\sin\theta }$$

where λ is the wavelength of the X-ray source (0.15418 nm) and θ is the angle of reflection (half of 2θ of the peak position). The d-spacing was calculated for the three most intense diffraction peaks, corresponding to the (111̅)/(002), (012) and (110) planes. In Figure 7, the change in the d-spacing of both studied polymers as a function of degradation time under various conditions is presented. As is presented, the only visible change in this structural parameter was observed for samples degraded by artificial weathering. The presented results suggest the insignificant ordering of the crystalline structures during degradation, which corresponds to the results of the macroscopic changes observed in the materials, e.g., stress and elongation of the sample. The studied materials became fragile after artificial weathering without significant increases in mass loss. Probably, the effect of artificial rainfall causes hydrolytic degradation, but the more important factors involved in polymer degradation are the temperature and exposure to UV radiation, which may support the crystallization and ordering of polymer chains [41].

## 4. Conclusions

The performed investigations on the degradation of a commercially available PBS and PBSA copolymer in various environments affords a better understanding of the changes in the polymers, not only on a macroscale, but also on molecular and supramolecular scales. The results of the macroscopic measurements confirmed the better degradability of the PBSA copolymer compared with the PBS homopolymer. The most favorable degradation ENVIRONMENT was compost, which contains microorganisms and natural enzymes that support degradation.

The analysis of the molecular parameters and supramolecular structures of both polymers during degradation showed that more significant changes occurred for PBSA. All of the applied environments resulted in a decrease in the molar mass and an increase in the crystallinity of both polymers, but larger changes were recorded for PBSA.

The analysis of the dispersity showed that each of the selected environments involved a different mechanism for the molecular changes and degradation. In the case of artificial weathering, the degradation of the polymer chains is random, and the dispersity increases with decreasing molar mass. The obviously different mechanism of chain decomposition is observed during biodegradation in compost, where the relatively slow hydrolysis of higher molar mass polymers and faster enzymatic etching of oligomers was observed by the decrease in molar mass with decreasing dispersity. Different results were recorded during degradation in soil, where only the molar mass was changed without changes in the dispersity.

The investigation of the supramolecular changes clearly showed the ordering of polymeric chains during degradation, as measured by the d-spacing. The most visible ordering was observed for the samples degraded by artificial weathering. In this case, the changes were more visible in PBS than in the PBSA copolymer.
